# Effect of the molecular adsorbent recirculating system and Prometheus devices on systemic haemodynamics and vasoactive agents in patients with acute-on-chronic alcoholic liver failure

**DOI:** 10.1186/cc4985

**Published:** 2006-07-19

**Authors:** Wim Laleman, Alexander Wilmer, Pieter Evenepoel, Ingrid Vander Elst, Marcel Zeegers, Zahur Zaman, Chris Verslype, Johan Fevery, Frederik Nevens

**Affiliations:** 1 Department of Hepatology, University Hospital Gasthuisberg, KU Leuven, Belgium; 2 Department of Medical Intensive Care, University Hospital Gasthuisberg, KU Leuven, Belgium; 3 Department of Nephrology, University Hospital Gasthuisberg, KU Leuven, Belgium; 4 Department of Laboratory Medicine, University Hospital Gasthuisberg, KU Leuven, Belgium

## Abstract

**Introduction:**

Patients with acute-on-chronic liver failure show an aggravated hyperdynamic circulation. We evaluated, in a controlled manner, potential changes in systemic haemodynamics induced by the molecular adsorbent recirculating system (MARS) and the Prometheus system liver detoxification devices in a group of patients with acute-on-chronic liver failure.

**Methods:**

Eighteen patients (51.2 ± 2.3 years old; Child–Pugh score, 12.5 ± 0.2; Maddrey score, 63.1 ± 5.0; hepatic venous pressure gradient, 17.6 ± 0.9 mmHg) with biopsy-proven alcoholic cirrhosis and superimposed alcoholic hepatitis were either treated with standard medical therapy (SMT) combined with MARS (*n *= 6) or Prometheus (*n *= 6) or were treated with SMT alone (*n *= 6) on three consecutive days (6 hours/session). Liver tests, systemic haemodynamics and vasoactive substances were determined before and after each session.

**Results:**

Groups were comparable for baseline haemodynamics and levels of vasoactive substances. Both MARS and Prometheus decreased serum bilirubin levels (*P *< 0.005 versus SMT), the Prometheus device being more effective than MARS (*P *= 0.002). Only MARS showed significant improvement in the mean arterial pressure (Δchange, +9 ± 2.4 mmHg versus -0.3 ± 2.4 mmHg with Prometheus and -5.2 ± 2.1 mmHg with SMT, *P *< 0.05) and in the systemic vascular resistance index (Δchange, +131.5 ± 46.2 dyne.s/cm^5^/m^2 ^versus -92.8 ± 85.2 dyne.s/cm^5^/m^2^with Prometheus and -30.7 ± 32.5 dyne.s/cm^5^/m^2 ^with SMT; *P *< 0.05), while the cardiac index and central filling remained constant. This circulatory improvement in the MARS group was paralleled by a decrease in plasma renin activity (*P *< 0.05), aldosterone (*P *< 0.03), norepinephrine (*P *< 0.05), vasopressin (*P *= 0.005) and nitrate/nitrite levels (*P *< 0.02).

**Conclusion:**

The MARS device, and not the Prometheus device, significantly attenuates the hyperdynamic circulation in acute-on-chronic liver failure, presumably by a difference in removal rate of certain vasoactive substances. These findings suggest conspicuous conceptual differences among the albumin dialysis devices.

## Introduction

The natural course of chronic liver disease is often complicated by acute episodes of potentially reversible decompensation, triggered by a precipitating event such as infection or upper gastrointestinal bleeding, and is frequently referred to as acute-on-chronic liver failure (AoCLF) [1-3]. This complication is associated with a further aggravation of the systemic haemodynamic dysfunction associated with portal hypertension, also called the hyperdynamic state. The hyperdynamic state is characterized by a reduced systemic vascular resistance and mean arterial pressure (MAP), as well as an increased cardiac index, heart rate and total plasma volume.

The pathophysiology is still incompletely understood but an overload of endogenous vasodilatory substances, including nitric oxide (NO), are presumed to play a major role [4-6]. The combination of vasodilatation and expanded intravascular volume is associated with abnormal distribution of the plasma volume with hypervolaemia in the splanchnic region (splanchnic hyperaemia) and other noncentral vascular territories, while relative hypovolaemia prevails in the central thoracic compartment. Sensor mechanisms interpret this relative central hypovolaemia as a reduced effective circulating volume, which results in an activation of endogenous vasoconstrictor and water-retaining and sodium-retaining systems such as the renin–angiotensin–aldosterone system and the sympathic nervous system, and in the nonosmotic release of arginine vasopressin. Persistent activation of these systems appears to worsen the increased intrahepatic resistance, thus playing a central role in the aggravation of portal hypertension, and promotes vasoconstriction with ischaemia in essential organs, which may lead to the development of the hepatorenal syndrome, portopulmonary hypertension and, finally, multiorgan failure [6-10]. Several studies have emphasized the relation between the degree of arterial hypotension in cirrhosis and the severity of portal hypertension, hepatic dysfunction, signs of decompensation and survival [8,11,12].

AoCLF is at present managed by treating the precipitating event and offering supportive therapy for end-organ dysfunction in the hope that liver function recovers. Unfortunately, 30–50% of these patients will die within 30 days [13].

**Figure 1:**
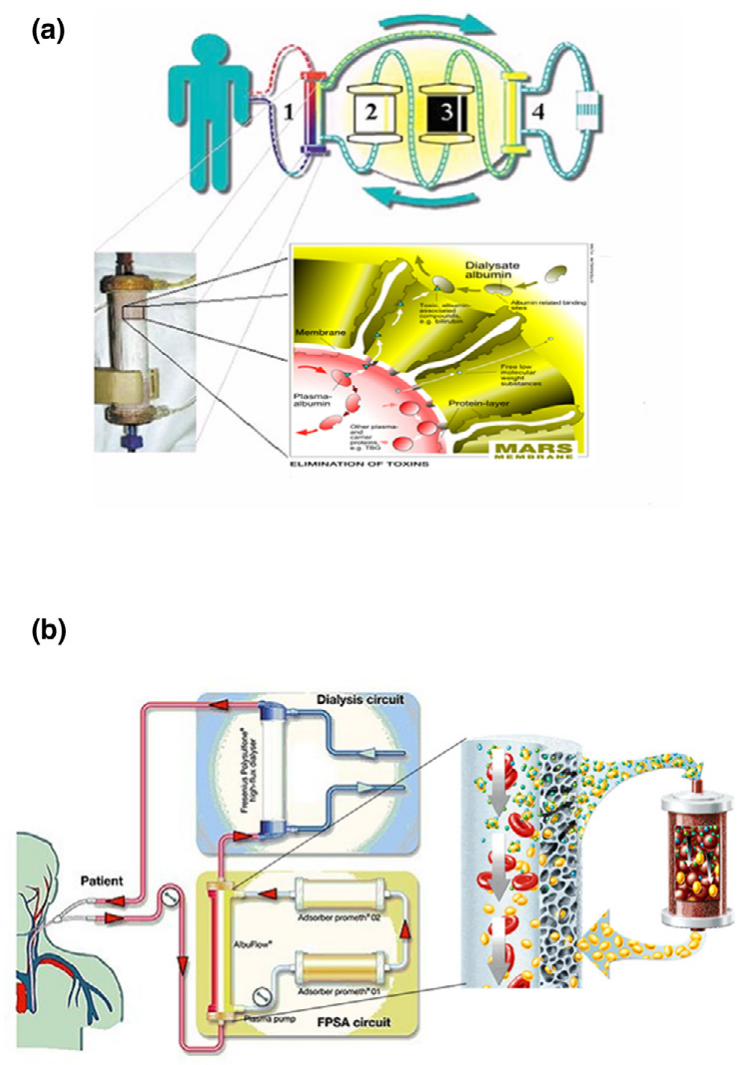
Schematic representation of both liver assist devices: **(a) **the molecular adsorbent recirculating system and **(b) **the Prometheus system. FPSA, fractionated plasma separation, adsorption, and dialysis.

Extracorporeal liver detoxification devices such as the molecular adsorbent recirculating system (MARS) [14] and fractionated plasma separation, adsorption, and dialysis (FPSA) – the Prometheus system [15] – have recently been proposed as novel treatment options for severe liver failure and its complications (Figure 1). For the MARS device, besides detoxification, several studies have ascribed a beneficial effect on portal and/or systemic haemodynamics [3,16-21]. In contrast, the impact of the Prometheus device on these variables remains currently unknown. We therefore aimed to compare, in a controlled manner, potential changes on systemic haemodynamics induced by the MARS or Prometheus system in a group of patients with alcoholic AoCLF and concomitant hyperdynamic circulation, and to evaluate associated changes in vasoactive substances.

## Methods

After obtaining approval of the Medical Ethics Committee of the University Hospital Gasthuisberg, signed informed consent was obtained from all patients or through their next of kin.

### Patient selection

#### Inclusion criteria

The criteria for study entry were histologically (transjugular liver biopsy) proven alcoholic cirrhosis with superposed alcoholic hepatitis, portal hypertension with associated hyperdynamic circulation (assessed by the hepatic venous pressure gradient and invasive haemodynamic monitoring), and AoCLF (defined as a persistent deterioration in liver function despite treatment of the precipitating event and characterized by an elevated bilirubin above 12 mg%).

#### Exclusion criteria

Patients were excluded if they were younger than 18 years or older than 75 years old, if they had evidence of extrahepatic cholestasis, of coma of nonhepatic origin or of active gastrointestinal bleeding in the past five days before inclusion, if they had comorbidities associated with poor outcome (necrotic pancreatitis, neoplasia, severe cardiopulmonary disease defined by a New York Heart Association score >3, or oxygen-dependent or steroid-dependent chronic obstructive pulmonary disease), and if there was clinical or microbiological evidence (culture of urine, blood, sputum and ascites) indicative of ongoing infection. For the purpose of this study, patients with hepatorenal syndrome type I were excluded since in our unit these patients are routinely treated with terlipressin and albumin, which might have interfered with the haemodynamic measurements.

### Study design

The study was designed to include 18 patients, who were randomly assigned (by the method of sealed envelopes) to either standard medical therapy (SMT) or to SMT with additional extracorporeal treatment (MARS or Prometheus device). Treatment was initiated the day after randomization and was performed during three successive days with a duration of six hours for each session.

### Standard medical therapy

SMT consisted of antibiotic therapy given as primary prevention of spontaneous bacterial peritonitis in patients with tense ascites. Additionally, in these patients sodium chloride was restricted to <4 g/day. In case of diuretic-resistant or impaired respiratory function, therapeutic paracentesis with simultaneous intravenous infusion of albumin (8 g/l ascites upon removal of more than 5 l) was administered. Lactulose was given in patients with encephalopathy, and if the encephalopathy worsened under this regime the protein intake was reduced to 0.5 g/kg/day.

Vitamin K was given at 10 mg/day intravenously. Substitution of fresh frozen plasma or appropriate amounts of packed cells was instated when the prothrombin time dropped below 10% and haemoglobin decreased below 8 g/dl, respectively. Substitution of fresh-frozen plasma, packed cells, human serum albumin or any other plasma expander was recorded. No compensation was foreseen for asymptomatic hyponatraemia above 120 mEq/l. High caloric nutrition with carbohydrates, lipids and proteins was calculated at 25 kcal/kg body weight/day in order to deliver a maximum amino acid intake of 1.2 g/kg body weight/day. None of the patients were treated with corticosteroids or experimental drugs during the study period.

### Extracorporeal liver assist

Vascular access was obtained for both the MARS and Prometheus devices with a dual-lumen haemodialysis catheter (Baxter, Brussels, Belgium), preferably placed in a femoral vein.

#### Molecular adsorbent recirculating system

The MARS device (kindly provided by Dirinco, Rosmalen, The Netherlands) consisted of a standard dialysis machine (AK100; Gambro, Leuven, Belgium) to drive the extracorporal blood circuit and an extra device to run and monitor a closed-loop albumin circuit (MARS Monitor; Teraklin AG, Rostock, Germany) (Figure 1a). The blood flow rate was 150–200 ml/minute. An albumin-impregnated, highly permeable dialyser (MARS-flux; Teraklin AG) was used. The closed-loop dialysate circuit was primed with 600 ml of 20% human serum albumin (CAF-DCF, Brussels, Belgium) and was driven by a roller pump of the MARS monitor at 200 ml/minute. The closed-loop dialysate is regenerated online by dialysis against a bicarbonate-buffered dialysate (processed by the dialysis machine), followed by passage through a column with uncoated charcoal and through a second column with an anion exchanger resin adsorber.

Anticoagulation was carried out with unfractionated heparin as a priming dose in the arterial line of the extracorporeal circuit followed by a maintenance infusion, with dosage adjustments to maintain the activated clotted time between 150 and 200 s.

#### The Prometheus system

The Prometheus system (Figure 1b) combines FPSA with high-flux haemodialysis for the removal of both albumin-bound and water-soluble toxins. The clearance of toxins is achieved in several steps. Firstly, albumin is separated from blood by a novel capillary albumin dialyser (AlbuFlow; Fresenius Medical Care AG, Bad Homburg, Germany). The AlbuFlow is made of polysulfone hollow fibres and is permeable to albumin (sieving coefficient, 0.6), and hence to albumin-bound substances. The blood flow rate was 150–200 ml/minute. The albumin filtrate in the FPSA circuit is subsequently perfused through a column with neutral resin (prometh01) and through a second column with an anion exchanger resin adsorber (prometh02), whereby the bound toxins are captured by direct contact with the high-affinity adsorbing material. The 'native' albumin is then returned to the patient. The FPSA recirculation circuit is driven by a roller pump at 300 ml/minute. Finally, after passage through the AlbuFlow, the patient's blood is dialysed through a high-flux dialyser (FX50; Fresenius Medical Care AG, Bad Homburg, Germany), whereby water-soluble toxins are eliminated. Maintenance and monitoring of the extracorporeal circuit is performed by a modified 4008 H haemodialysis unit Fresenius Medical Care AG, Bad Homburg, Germany).

Anticoagulation was achieved by either unfractionated heparin (priming dose followed by a maintenance infusion) or regional citrate.

### Monitoring procedures

#### Biochemical measurements

Serum values with relation to inflammation (leukocytosis and C-reactive protein), to excretory and desintoxication function (ammonia, bile acids, bilirubin), to synthesis capacity (albumin, prothrombin time), to renal function (creatinine, ureum, sodium) and to endogenous vasopressors (plasma renin activity, aldosterone, vasopressin and catecholamines) were measured using standard laboratory procedures. Serum samples were also assayed for nitrate/nitrite (NOx), a parameter of NO production, via a fluorometric method using 2,3-diaminonaphtalene as previously described with slight modifications [22].

At the end of the sessions, dialysate samples were taken to determine potential elimination of neurohumoral pressor systems and NOx. More specifically, for MARS the dialysate was sampled with a syringe from the closed loop circuit immediately after the MARS flux, whereas in the PROMETHEUS system the equivalent location was immediately after passage through the AlbuFlow. If elimination was found, the treatment phase percentage reduction rate (RR_T_) was calculated: RR_T _= 100 × (1 - C_after_/C_before_), with C_after _and C_before _being the serum level before and after treatment, as described elsewhere [23,24].

All samples were taken in a supine position and at the same time as the haemodynamic measurements were done, in order to guarantee reproducible time-points.

#### Haemodynamic measurements

A radial artery catheter was used for monitoring the MAP, the heart rate and blood sampling. For measurements of the central venous pressure, the cardiac index, the stroke volume and the systemic vascular resistance index (SVRI), either a four-lumen balloon-tipped, thermodilution, pulmonary artery catheter (Swan-Ganz; Baxter) was used or a pulse contour continuous cardiac output catheter (PiCCO; Pulsion Medical Systems AG, Munich, Germany) was used. It is currently accepted that measurement with this aortic transpulmonary thermodilution technique gives continuous and intermittent values that agree with the pulmonary thermodilution method, but in a less invasive manner [25,26].

Haemodynamic measurements were performed one hour prior to the MARS/Prometheus treatment and one hour after the end of the sessions. For the SMT group, haemodynamic measurements were carried out in the same time window as the SMT with MARS/Prometheus treatment group.

### Statistical analysis

Data are expressed as the mean ± standard error of the mean, and as Δchange ± standard error of the mean. The paired *t *test was used for pairwise comparison within groups, or the Wilcoxon test when appropriate. Analysis of variance was used for comparison between groups. Fisher's Exact test was used for comparison of frequencies. Data were analysed using SigmaSTAT 2.0 (Jandel Corporation, San Rafael, CA, USA). *P *≤ 0.05 was considered statistically significant.

## Results

### Patient demographics

Of a total of 35 patients with AoCLF eligible for potential liver support therapy, 18 patients were finally included in the study. Seventeen patients were excluded because of diverse reasons: nonalcoholic origin of AoCLF (eight patients), enrolment in another (multicentre) study (three patients), sepsis (two patients), hepatorenal syndrome type I (two patients), newly diagnosed cholangiocarcinoma (one patient) and fatal cardiac arrest before entry in the study (one patient). General characteristics of the included patients at study entry for each designated treatment group are presented in Table 1.

Transjugular liver biopsy in all patients, performed in the screening period, was consistent with the diagnosis of alcoholic cirrhosis with superimposed active alcoholic hepatitis. Leukocytosis and an elevated C-reactive protein level further emphasized the inflammatory nature of this condition (Table 2). In five patients variceal bleeding (*n *= 3) and spontaneous bacterial peritonitis (*n *= 2) were found as additional precipitating factors to alcoholic hepatitis at admission. In order to minimize the possibility of spontaneous improvement of liver function, a time interval, varying from 5 to 11 days, was respected between control of the precipitating event and inclusion into the study.

There were no differences between the three groups with regard to age, biochemical prognostic markers, hepatic venous pressure gradient, presence of ascites, grade of encephalopathy and different risk scoring systems for mortality. All patients were in an advanced state of decompensated liver disease, with a mean Sequential Organ Failure Assessment score and Model for End-stage Liver Disease score, respectively, of 8.9 (range, 6–12) and 25.6 (range, 16–35). These scores are associated with a mean three month predicted mortality between 76% and 88% [12,27]. The mean Maddrey score was 63.1, which implies a mortality risk above 50% in the absence of intervention [28].

### Biochemical analysis with regard to hepatic detoxification, synthetic liver capacity, systemic inflammation and renal function

Biochemical variables before and after treatment with MARS or Prometheus and SMT or with SMT alone are presented in Table 2. Baseline values of all groups were comparable.

**Figure 2:**
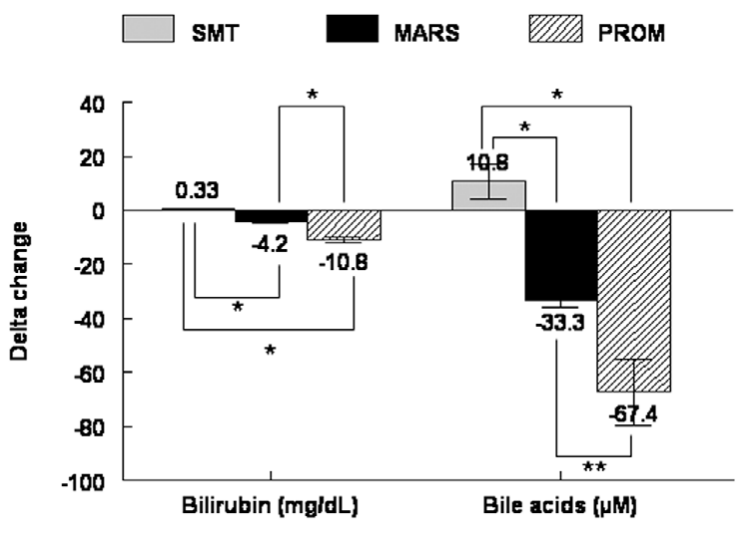
Comparison of changes in levels of bilirubin and bile after treatment with standard medical therapy (SMT) alone, with the molecular adsorbent recirculating system (MARS) or with Prometheus (PROM). *P < 0.001, **P < 0.05.

Levels of bilirubin and bile acids, representative of albumin-bound toxins, were significantly decreased after both MARS and Prometheus therapy (Table 2). The removal of these substances was more pronounced with the Prometheus device (Figure 2).

Re-evaluation of bilirubin levels 3 and 7 days after termination of the treatment period showed a comparable bilirubin level in the MARS-treated group at day three post-treatment (19.8 ± 3.8 mg% versus 16.6 ± 1.2 mg% at the end of treatment, *P *= 0.455), which increased to 25.3 ± 5 mg% at day 7 (*P *= 0.042 versus end of treatment). A similar evolution was noted in the Prometheus group – day 3 bilirubin levels amounted to 19.2 ± 3.3 mg% (versus 18 ± 3.2 mg% at the end of treatment, *P *= 0.590) and rose further to 26.3 ± 2.8 mg% at day 7 (*P *= 0.046 versus the end of treatment). In the SMT group, bilirubin remained stable at 29.1 ± 4.5 mg% on day 3 (*P *= 0.396 versus end of treatment) and 28 ± 5.4 mg% on day 7 (*P *= 0.840).

### Systemic haemodynamics

The baseline measurements were comparable between all groups. All patients showed hyperkinetic circulation with a low MAP (69.7 ± 1.1 mmHg), an elevated cardiac index (4.8 ± 0.3 l/minute/m^2^) and a low SVRI (1085 ± 76 dyne.s/cm^5^/m^2^). In the control group treated only with SMT, patients showed a further decrease in MAP (*P *= 0.05) without changes in the heart rate, the central venous pressure, the cardiac index and the SVRI (Table 3). After MARS treatment, we observed an improvement in the MAP (*P *= 0.014), stroke volume (*P *= 0.028) and SVRI (*P *= 0.036), whereas patients treated with Prometheus system showed stable haemodynamic parameters, except for an increased heart rate at the end of treatment (*P *= 0.001).

**Figure 3:**
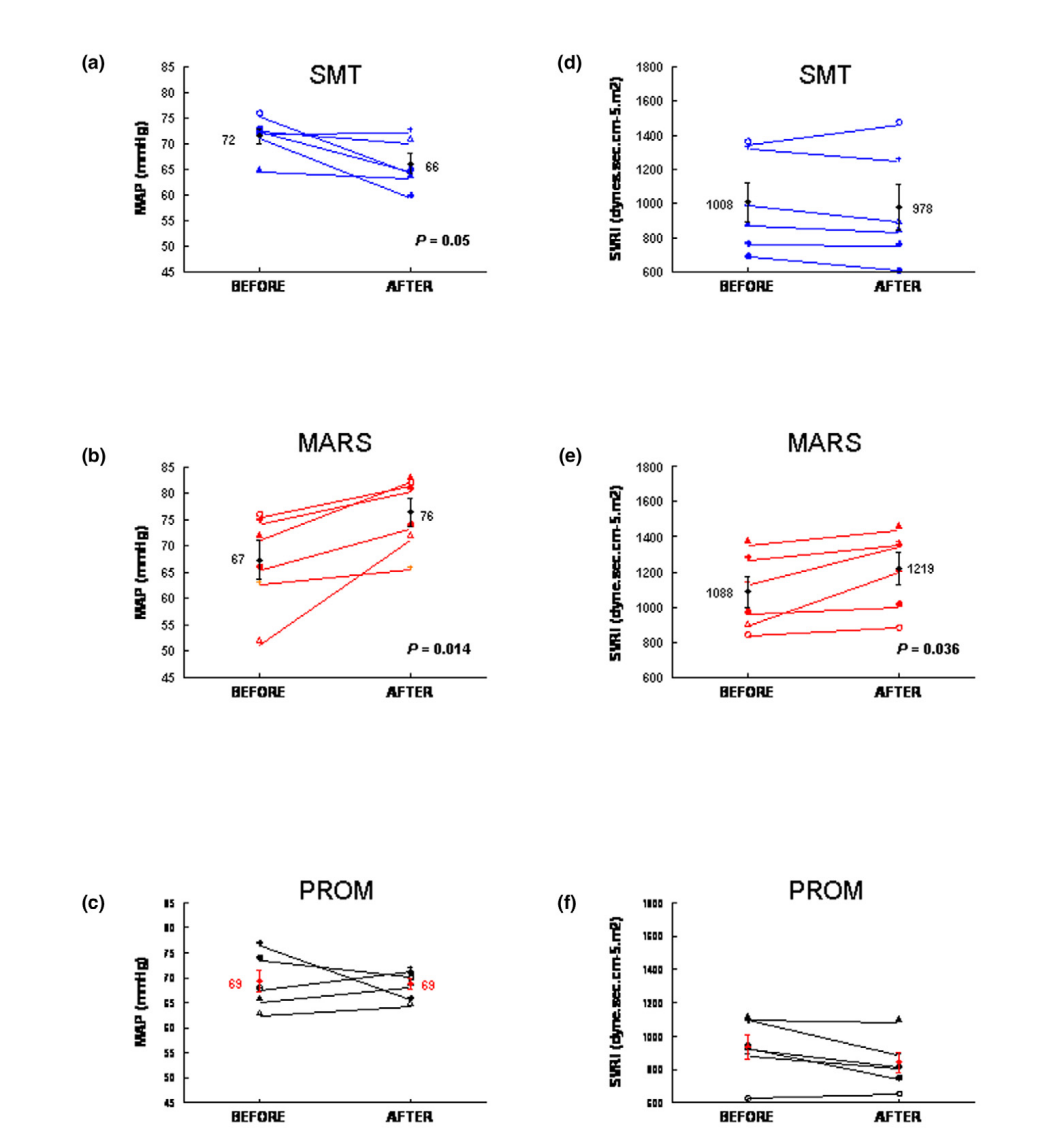
Individual values for the mean arterial pressure (MAP) and systemic vascular resistance index (SVRI) before versus after treatment with **(a)**, **(d) **standard medical therapy alone (SMT), **(b)**, **(e) **the molecular adsorbent recirculating system (MARS) and **(c)**, **(f) **the Prometheus (PROM) system. *P *value given when significant.

**Figure 4:**
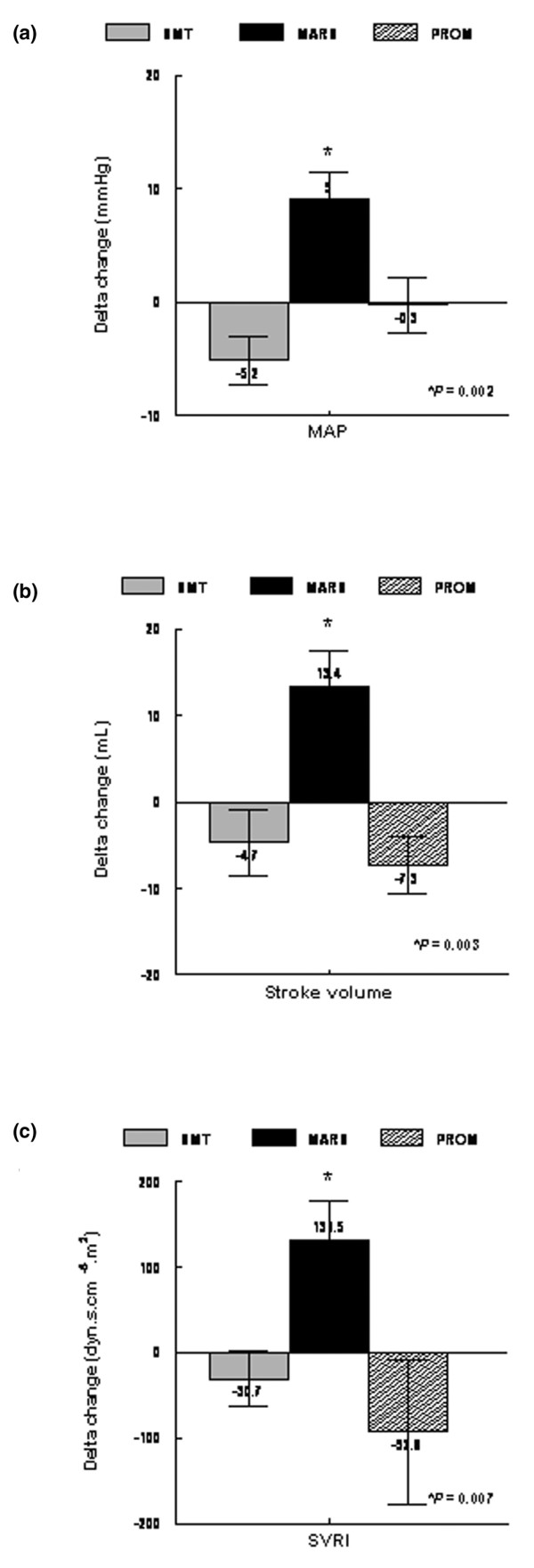
Comparison of changes in the mean arterial pressure (MAP), stroke volume and systemic vascular resistance index (SVRI) after treatment with standard medical therapy alone (SMT), with the molecular adsorbent recirculating system (MARS) or with the Prometheus (PROM) system. *P value given when significant.

Values of the MAP and the SVRI for individual patients are illustrated in Figure 3a–f. When the effect of treatment on systemic haemodynamics was compared between the different groups, the beneficial effect of MARS on the MAP (*P *= 0.002), on the stroke volume (*P *= 0.003) and on the SVRI (*P *= 0.007) was confirmed (Figures 4a–c). There was no significant change in the central venous pressure within or between the groups, suggesting maintenance of the circulating volume irrespective of extracorporeal liver support. The amount of packed cells, human serum albumin or any other plasma expander did not differ between groups. No significant difference was seen in urine production in the groups and between all groups over the study period.

Re-evaluation of the MAP three and seven days after termination of the treatment period showed a comparable MAP at day three post-treatment (74 ± 3 mmHg versus 76 ± 4 mmHg at the end of treatment, *P *= 0.807) which afterwards decreased to 66 ± 2 mmHg at day seven (*P *= 0.047 versus end of treatment). No differences in MAP were noted in the Prometheus and SMT group at day three and seven after the end of the study period compared with at the end of treatment.

### Endogenous vasoactive substances

**Figure 5:**
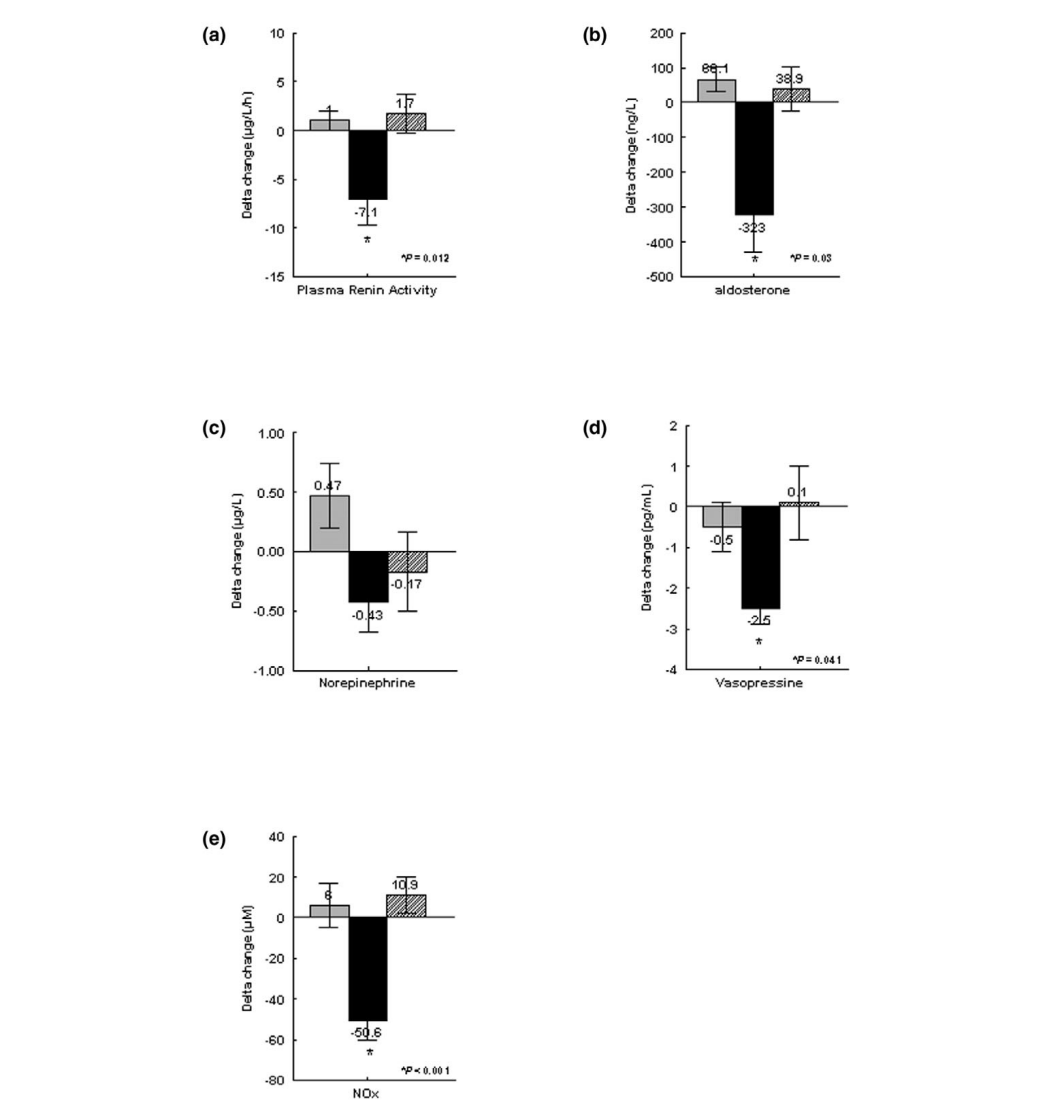
Comparison of changes in **(a) **plasma renin activity, **(b) **aldosterone, **(c) **norepinephrine, **(d) **vasopressin and **(e) **nitric oxide metabolites (NOx) after treatment with standard medical therapy (SMT) alone (grey shading) shading), with the molecular adsorbent recirculating system (MARS) (black shading) or with the Prometheus (hatched shading) (PROM) system shading). *P value given when significant.

Changes in levels of endogenous vasoactive substances within and between the different treatment groups are presented in Table 4 and Figure 5a–e, respectively. Baseline values of all groups were comparable. Similar to the direct haemodynamic assessment, we only observed changes in the MARS-treated group. All circulating endogenous vasopressors, including the renin–angiotensin–aldosterone system (plasma renin activity, *P *= 0.044; aldosterone, *P *= 0.027), norepinephrine (*P *= 0.043) and vasopressin (*P *= 0.005), decreased significantly after MARS treatment as compared with the group treated with Prometheus and SMT (Table 4). Additionally, NOx levels (metabolites of NO) were exclusively lowered in the MARS group (*P *= 0.012) (Table 4).

To evaluate potential elimination by the devices, circulating endogenous vasopressors and NOx levels were also measured in the albumin dialysate of both the MARS and Prometheus devices. Determination of the concentration of vasopressin in the dialysate was technically not possible due to a low detection window. Renin was undetectable in the dialysate of the closed loop of the MARS system. Aldosterone, norepinephrine and NOx were detected at concentrations of 363 ± 73 ng/l, 0.021 ± 0.05 μg/l and 54.9 ± 11.2 μM, respectively. The calculated RR_T _values of aldosterone, norepinephrine and NOx amounted to 59.5 ± 9%, 38.5 ± 8.3% and 53.4 ± 6.9%, respectively. In the MARS-treated group, a negative correlation was found between Δchange in the SVRI and in serum NOx levels (*R *= -0.943, *P *= 0.016).

In contrast to the MARS system, renin was detected in the dialysate of the Prometheus device (7.3 ± 1.9 μg/l/hour, RR_T _= -22.8 ± 25.1%). Aldosterone, norepinephrine and NOx levels were detected, respectively, at 80 ± 48.1 ng/l, 0.84 ± 0.3 μg/l and 20.9 ± 6.7 μM in the dialysate. The respective calculated RR_T _values were 11.4 ± 14.7%, -9.8 ± 17.2% and -15.9 ± 14.8 %.

### Outcome and safety profile

Overall, the Prometheus and MARS treatment devices were well tolerated.

In the MARS group, one patient developed trombocytopaenia with diffuse petechiae and a large haematoma in the left groin 3 days after ending MARS treatment, which after extensive investigation was attributed to alloimmunization. The patient recovered fully.

With the Prometheus device, we observed an episode of serious thrombocytopenia (125 × 10^-9^/l to 45 × 10^-9^/l) and hypercalcaemia in one patient, a one-time clotting of the secondary circuit in a second patient, and an episode of hypotension at the start of the procedure in a third patient.

## Discussion

The current concept of the pathogenesis of circulatory dysfunction in cirrhosis is based on the peripheral vasodilation hypothesis [4,6-8,29]. This hypothesis proposes that splanchnic arterial vasodilation is the initiating factor in the systemic haemodynamic dysfunction. At the early stages of disease, there is a homeostatic increase in the cardiac output as a result of the decrease in cardiac afterload and the stimulation of the sympathetic nervous system. With progression of the disease and intensified splanchnic vasodilation and subsequent systemic vasodilation, the cardiac compensation is no longer sufficient to balance the decreasing afterload. The arterial pressure decreases, which leads to a baroreceptor-mediated stimulation of sympathetic nervous activity and of the renin–angiotensin–aldosterone system, and to an increased nonosmotic release of vasopressin in an attempt to preserve circulatory homeostasis. Activation of these systems leads to renal salt and water retention, and to ascites. Additionally, the activation is also responsible for a further aggravation of the active intrahepatic vascular resistance and the development of multiorgan failure in cirrhosis [6,8,30].

The exact underlying pathophysiological mechanism to the hyperdynamic state remains unknown but probably represents a multifactorial phenomenon and may involve impaired neurogenic responses, accumulation of vasodilators and diminished responsiveness to a variety of vasoconstrictors [31]. Ample evidence suggests a central role for excessive production of NO in both the splanchnic and systemic vascular territories [31-36].

In the present study we observed an improvement of the hyperdynamic state during MARS therapy. It is unlikely that this haemodynamic improvement in the MARS group was induced by changes in fluid balance. Indeed, there were no differences between the treatment groups with regard to central filling, serum albumin and creatinine levels. In both dialysis groups, pump speeds and fluid exchange rates were similar. Furthermore, the favourable effect on the MAP in patients treated with MARS disappeared within four days after cessation of treatment, which further emphasizes a causal relationship to MARS. The improvement in the SVRI and MAP therefore strongly suggests temporary changes and/or elimination in endogenous vasoactive substances.

We observed a reduction in NOx levels that correlated negatively with the improvement in the SVRI. Additionally, this was associated with a drop in endogenous vasopressor systems, which are considered to act as counteracting factors for excessive splanchnic and systemic vasodilation, most probably induced by NO. The question of whether NO is directly removed from the circulation by the MARS device is difficult to explore as the metabolic fate of NO is still poorly understood. NO is a labile species with a half-life of only a few seconds [31]. NO therefore acts locally and is degraded or converted rapidly into intermediate metabolites. Putative intermediate metabolites include an array of low molecular weight and high molecular weight thiols, nitrosoglutathione, nitrosohaemoglobin and nitrosoalbumin, some of which might be present in sufficient quantities to exert biological effects [37-41]. Whether S-nitroso adducts of serum albumin act as a 'haemodynamically active' circulating depot of NO *in vivo*, as suggested by Stamler and colleagues [38] and Rafikova and colleagues [39], and result in the removal of NO in this way by MARS, remains subject to practical and theoretical criticism [38-41]. Furthermore, the value of measuring NOx as a parameter of NO synthase activity can also be discussed, but at present it remains by far the most 'simple' and direct index of NO generation [37].

A second explanation of how MARS might affect the systemic characteristics of portal hypertension is that it might directly target the active, modifiable component of the increased intrahepatic vascular resistance by a decrease in the intrahepatic action of vasoconstrictors [16,42]. In the present study we found a reduction in vasopressor hormones during MARS treatment, suggesting either decreased production or elimination, or a combination of both. Combined elimination and decreased production seems most probable. We could demonstrate the presence of pressor hormones, such as norepinephrine and aldosterone, in the closed loop albumin dialysate, whereas renin was undetectable in dialysate samples despite decreased serum levels after MARS treatment. Because of its molecular size, renin is not removable by MARS, indicating that MARS exerts control on the degree of counteractivation of endogenous vasoconstrictor systems. This latter observation suggests that the reduction in vasopressors is a consequence of the improved haemodynamic situation, therefore favouring the hypothesis of primarily eliminating a systemic vasodilating substance, such as NO.

A third possibility involves removal of inflammatory mediators due to the inflammation-related precipitant of the AoCLF in all of these patients, more specifically alcoholic steatohepatitis. Tumour necrosis factor alpha, a highly expressed proinflammatory cytokine, particularly in alcoholic steatohepatitis, is currently considered the most important vasoactive inflammatory mediator in this context [43]. More specifically, it has been shown that antagonism of tumour necrosis factor alpha with anti-tumour necrosis factor alpha antibody attenuates portal hypertension and the associated hyperdynamic state, both experimentally [44] and in humans [45].

Sen and colleagues [46] studied the effect of MARS on tumour necrosis factor alpha in patients with alcoholic AoCLF and found no changes in plasma levels after seven days of MARS treatment, despite a documented removal of tumour necrosis factor alpha and its receptor TNF-R1, suggesting that the concurrent cytokine production due to the disease process itself balanced any removal. In the current study, inflammatory markers, such as leukocytosis and C-reactive protein, remained unchanged in all groups, which supports the findings of Sen and colleagues [16,46]. Together these data re-emphasize the hypothesis that MARS removes a putative circulating vasoactive circulating factor from the peripheral blood independent of changes in vasoactive cytokines.

Our results further suggest that MARS is effective in temporarily improving haemodynamics, while the Prometheus system does not. In contrast, the Prometheus device showed a higher ability of clearing albumin-bound toxins [24,47] than MARS. A possible explanation for this finding relates to conceptual differences (Figure 1). The Prometheus device is based on FPSA, which involves selective filtration of the native albumin ('fractionated plasma separation') through a specific albumin-permeable polysulfon filter (with a cutoff value of 250 kDa) into a secondary circuit. In this secondary circuit, the albumin fraction is then directly purified by adsorption on a neutral resin adsorber and an anion exchanger, and thereafter returned to the plasma. In addition to the FPSA step, high-flux haemodialysis is performed. The filtration step presumably is the predominant factor in the lack of haemodynamic effects [48].

Besides a decreased potential to eliminate the investigated endogenous vasoactive substances, as documented by the lower calculated reduction ratios for these hormones compared with those of MARS, the FPSA step of the Prometheus system might also have been responsible for minimal changes in colloid osmotic pressure during the procedure. These minimal changes could have caused subclinical volume shifts that prohibited the deactivation of the homeostatic baroreceptor response [15]. Indeed, in our study the concurrent production of endogenous vasoactive substances itself balanced any removal since no differences in the levels of these agents were observed at the end of treatment compared with the beginning of treatment. The increased heart rate, as a compensatory mechanism, might also be an indirect index of subclinical volume shifts.

Several factors make patients with AoCLF extremely sensitive to volume changes, such as the decreased effective circulating volume with persistent counteracting vasopressor response, the increased cardiac output with impaired end-diastolic filling and the autonomic vascular dysfunction [49].

## Conclusion

The present study demonstrates that MARS improves haemodynamic disturbances in patients with acute on chronic alcoholic liver failure with associated hyperdynamic circulation. These beneficial haemodynamic effects can be explained by the removal of circulating endogenous vasoactive substances and are in agreement with the current hypothesis of the pathogenesis of portal hypertension.

## Key messages

• 	Central in the pathogenesis of AoCLF, defective detoxification leads to life-threatening complications, including an aggravated hyperdynamic circulation.

• 	The liver assist devices, MARS and Prometheus, are extracorporeal, nonbiological liver support devices able to remove albumin-bound toxins, typically present in AoCLF.

• 	Compared with SMT alone, the MARS and Prometheus devices significantly decrease albumin-bound toxins (bile acids, bilirubin) in patients with alcoholic AoCLF.

• 	Additionally, the MARS device, but not the Prometheus device, also attenuates the hyperdynamic circulation by increasing both the MAP and SVRI, probably by the demonstrated decrease in endogenous circulating vasoactive substances. This further supports the crucial role of vascular mediators in the pathophysiology of portal hypertension in humans.

• 	The haemodynamic difference in this study emphasizes conspicuous conceptual differences among the albumin dialysis devices.

## Abbreviations

AoCLF = acute-on-chronic liver failure; FPSA = fractionated plasma separation, adsorption, and dialysis; MAP = mean arterial pressure; MARS = molecular adsorbent recirculating system; NO = nitric oxide; NOx = nitrite/nitrate; RR_T _= treatment phase percentage reduction rate; SMT = standard medical therapy; SVRI = systemic vascular resistance index.

## Competing interests

WL was supported by a grant offered by the Fund for Scientific Research (Aspirant mandaat – FWO Vlaanderen). FN was supported by FWO Vlaanderen G.0495.04.

## Authors' contributions

WL, AW and FN were involved in the design, performance, coordination and statistical analysis of the study as well as the drafting of the manuscript. PE, CV and JF participated in the design and helped to draft the manuscript. ZZ, IVE and MZ were involved in biochemical measurements and assisted in helpful discussions. All authors read and approved the final manuscript.

## Figures and Tables

**Table 1:** General characteristics of the different treatment groups

	Standard medical therapy (*n *= 6)	Molecular adsorbent recirculating system + standard medical therapy (*n *= 6)	Prometheus + standard medical therapy (*n *= 6)	*P *value
Age (years)	55.8 ± 1.9	54.5 ± 3.0	43.2 ± 4.7	0.097
Sex (male/female)	3/3	5/1	4/2	0.585
Serum bilirubin (mg%)	29.9 ± 4.5	25.6 ± 3.6	34.3 ± 1.9	0.245
Prothrombin time	37.6 ± 2.9	39.8 ± 4.0	40.9 ± 7.0	0.888
Albumin (g/l)	26.3 ± 1.7	27.8 ± 0.8	29.4 ± 1.7	0.348
Creatinin (mg%)	1.5 ± 0.3	1.6 ± 0.5	1.5 ± 0.2	0.984
Hepatic encephalopathy score	1 ± 0.4	1.5 ± 0.2	1.2 ± 0.4	0.473
Hepatic venous pressure gradient (mmHg)	16.8 ± 1.4	19.2 ± 1.0	15.5 ± 2.5	0.245
Child-Pugh score	13 ± 0.4	12.7 ± 0.3	11.8 ± 0.3	0.070
Sequential Organ Failure Assessment score	9.3 ± 0.9	8.3 ± 0.8	9.2 ± 0.7	0.637
Model for End-stage Liver Disease score	24.3 ± 2.4	22.7 ± 2.2	29.7 ± 1.7	0.081
Maddrey's discriminant function	70.5 ± 10.5	55.6 ± 7.3	63.2 ± 18.1	0.501

Data presented as the mean ± standard error of the mean, and represent values of the day before start of study.

**Table 2:** Biochemical parameters before and after three successive days of standard medical therapy (SMT) alone or treatment with the molecular adsorbent recirculating system (MARS)/Prometheus devices combined with SMT

	SMT		MARS + SMT		Prometheus + SMT	
	
	Before treatment	After treatment	Before treatment	After treatment	Before treatment	After treatment
Ammonia (μM)	65.6 ± 8.8	72.9 ± 9.2	57.8 ± 5.3	63.7 ± 11.1	45.4 ± 3.3	49 ± 7.4
Bilirubin (mg/dl)	29.5 ± 4.3	29.9 ± 4.4	21.8 ± 2.6	16.6 ± 1.2*	29.7 ± 2.9	18 ± 3.2**
Bile acids (μM)	162 ± 36.7	172.7 ± 34	111 ± 17.1	78 ± 19.7**	145.3 ± 16.4	78.1 ± 14.1**
Albumin (g/l)	29 ± 1.7	29.8 ± 1.8	27.7 ± 0.9	28.9 ± 1.3	25.7 ± 1.1	25.3 ± 1.9
Prothrombin activity (%)	36.8 ± 2.5	34.7 ± 3.4	39.9 ± 4.2	39.8 ± 4.2	37.3 ± 5.5	28.1 ± 6.1
Creatinin (mg/dl)	1.3 ± 0.4	1.4 ± 0.4	1.4 ± 0.3	1.1 ± 0.2	1.2 ± 0.1	0.9 ± 0.1
Urea (mg/dl)	66.7 ± 20	77.3 ± 21.5	64.1 ± 22	41.8 ± 13.2	51.5 ± 22.3	27.8 ± 10.3
Leukocyte count (× 10^9^/l)	12.6 ± 2.6	13.8 ± 2.9	13.8 ± 1.8	13.8 ± 1.7	19.5 ± 5.8	16.4 ± 2.9
C-reactive protein (mg/l)	33.2 ± 9.7	32.3 ± 9.8	53.5 ± 9.0	58 ± 7.4	41.2 ± 7.6	31.1 ± 6.7

Data presented as the mean ± standard error of the mean. *P *value versus start of the study period: *P < 0.05, **P ≤ 0.001.

**Table 3:** Haemodynamic parameters before and after three successive days of standard medical therapy (SMT) alone or treatment with the molecular adsorbent recirculating system (MARS)/Prometheus devices combined with SMT

	Normal range	SMT		MARS + SMT		Prometheus + SMT	
		
		Before treatment	After treatment	Before treatment	After treatment	Before treatment	After treatment
Heart rate (beats/min)	60–100	81 ± 3	81 ± 3	77 ± 7	83 ± 6	86 ± 5	97 ± 6*
Mean arterial pressure (mmHg)	72–102	72 ± 1	66 ± 2**	67 ± 4	76 ± 3**	69 ± 2	69 ± 1
Stroke volume (ml)	70–130	120 ± 16	115 ± 17	107 ± 18	120 ± 15**	107 ± 8	99 ± 11
Central venous pressure (mmHg)	1–9	11 ± 2	10 ± 2	9 ± 2	9 ± 2	10 ± 2	11 ± 2
Cardiac index (ml/min/m^2^)	2.5–4	5.1 ± 0.4	5 ± 0.4	4.2 ± 0.4	4.5 ± 0.4	5.3 ± 0.5	5.4 ± 0.6
Systemic vascular resistance index (dyn.s/cm^5^/m^2^)	1760–2600	1008 ± 116	978 ± 134	1088 ± 88.1	1219 ± 93**	934 ± 72	841 ± 62

*P *value versus start of the study period: *P ≤ 0.001, **P < 0.05.

**Table 4:** Evolution of endogenous vasoactive substances before and after standard medical therapy (SMT) alone or treatment with the molecular adsorbent recirculating system (MARS)/Prometheus devices combined with SMT

	SMT		MARS + SMT		Prometheus + SMT	
	
	Before treatment	After treatment	Before treatment	After treatment	Before treatment	After treatment
Plasma renin activity (μg/l/hour)	9.8 ± 3.0	10.8 ± 3.4	12.6 ± 3.9	5.5 ± 2.3*	10.7 ± 3.4	12.4 ± 4.2
Aldosterone (ng/l)	417.4 ± 102.1	483 ± 124.9	460.5 ± 123.2	137.5 ± 30.2*	394.4 ± 176.7	433.3 ± 235.4
Norepinephrine (μg/l)	0.90 ± 0.24	1.36 ± 0.47	0.82 ± 0.19	0.46 ± 0.07*	1.28 ± 0.43	1.31 ± 0.40
Vasopressin (pg/ml)	10.8 ± 1.7	10.2 ± 1.5	8.9 ± 0.6	6.5 ± 0.7**	9.4 ± 1.4	9.4 ± 1.3
Nitrate/nitrite (μM)	68.6 ± 9.9	74.7 ± 13.1	91.2 ± 16.1	40.4 ± 5.9*	73.6 ± 14.4	84.4 ± 17.6

*P *value versus start of the study period: *P < 0.05, **P ≤ 0.005.
